# PAD4 deletion in synovial macrophages exacerbates pathology in inflammatory arthritis

**DOI:** 10.21203/rs.3.rs-9653857/v1

**Published:** 2026-06-11

**Authors:** Maximilian G Mayr, Gaurav Gadhvi, Paul Thompson, Roman Fischer, Harris R Perlman, Deborah R Winter, Anna B Montgomery

**Affiliations:** Northwestern University; Northwestern University; University of Massachusetts Medical School; University of Oxford; Northwestern University; Northwestern University; King’s College London

**Keywords:** Macrophage, Citrullination, Inflammatory arthritis, PAD4

## Abstract

Citrullination by peptidylarginine deiminase (PAD) enzymes is a post-translational protein modification implicated in the etiopathogensis of rheumatoid arthritis. Of the five known PAD isoforms, PAD4 is expressed in the nucleus of innate immune cells including synovial macrophages, and studies have shown citrullination of intracellular proteins such as transcription factors and histones can polarize cell phenotype. Here, we report how systemic and local deletion of PAD4 in synovial macrophages from PAD4^−/−^ or PAD4^f/f^Cre^LysM^ mice results in activation of both shared and distinct gene modules in four populations of synovial macrophages. Furthermore, severity of KBxN serum transfer arthritis was increased in PAD4^f/f^Cre^LysM^ mice compared to controls. Furthermore, macrophages isolated from the hindjoints of arthritic PAD4^f/f^Cre^LysM^ displayed increased expression of inflammatory genes compared to macrophages from arthritic PAD4^−/−^ mice. In addition, we report that local intra-articular administration of PAD inhibitor BB-Cl-amidine increased arthritis severity, while systemic administration had no effect. These findings indicate an anti-inflammatory role for intracellular citrullination in synovial macrophages, and highlight the potential confounding effects of extracellular and systemic PAD4 loss of function.

## Background

Citrullination is the post-translational conversion of arginine to citrulline catalyzed by protein arginine deiminases (PADs). There are five PAD isoforms (PAD1–4, PAD6), but only PADs 1–4 are catalytically active. While PAD4 is the only PAD with a nuclear localization signal, PADs 1, 2, and 4 have all been shown to citrullinate histones and other nuclear proteins and as such can regulate transcription (1–3). Histone citrullination also controls the pluripotency of stem cells (4), and primes neutrophil extracellular trap (NET) formation in response to inflammatory stimuli (5). Notably, NETosis results in the release of PADs into the extracellular space, increasing citrullination to modify protease activity (6). Citrullination of transcription factors NF_K_B and E2F-1 also leads to increased expression of pro-inflammatory cytokines TNF and IL-1β in neutrophils (7, 8). In fact, citrullination of RNA-polymerase-II itself can regulate transcription (9).

Citrullination has been linked to RA primarily due to the presence of anti-citrullinated protein auto-antibodies (ACPA) in patients, that develop before disease onset (10). However, study of ACPA has contributed little to understanding the function of citrullination and inflammatory pathology in RA. Given levels of citrullination are increased in the synovial fluid and tissue of RA patients, it is possible increased citrullination has direct implications on disease pathology independent of ACPA (6, 11). Furthermore, a number of single nucleotide polymorphisms (SNPs) in the PAD4 encoding gene region are associated with development of RA, suggesting increased citrullination may provide a genetic contribution to disease etiology (12, 13).

In mouse models of arthritis, PAD4^−/−^ mice display reduced severity of collagen-induced arthritis (CIA), and tumor necrosis factor-α (TNF-α) induced arthritis compared to controls (14, 15). Furthermore, CIA severity was also reduced using the pan PAD inhibitors Cl-amidine and BB-Cl-amidine as well as the PAD4 selective inhibitor GSK199 (16, 17). By contrast, the pathology of serum transfer induced arthritis (STIA) was not affected in PAD4^−/−^ as measured by clinical score, swelling, joint erosion or joint invasion (18); however, levels of PAD4 gene *Padi4* were elevated in the spleen of STIA mice, suggesting citrullination is increased during inflammation and thus could contribute to disease pathology (19). These data support involvement of citrullination in the pathology of inflammatory arthritis, although specific mechanisms are yet to be investigated.

Synovial macrophages are a key effector cell mediating joint damage in inflammatory arthritis. Multiple factors can augment macrophage phenotype including origin, tissue location, transcriptional profile, and immune-metabolism. In a unique example of macrophage polarization in RA, binding of ACPA to uncharacterized citrullinated proteins on macrophages promotes an inflammatory macrophage phenotype through increased IRF5 expression (20). *In vitro*, differentiation of monocyte-derived macrophages in the presence of citrullinated fibrinogen polarized resulting macrophages to an increased inflammatory state (21). In addition, PAD expression has also been shown to increase in differentiated macrophages, compared to undifferentiated U937 cell lines (22). These data suggest citrullination can influence macrophage phenotype. To that end, in this study we investigated the effects of PAD4 deletion on synovial macrophages in the context of serum transfer induced arthritis (STIA). Deletion of PAD4 in steady-state resulted in increased expression of inflammatory genes in synovial macrophages, which was exacerbated in STIA and contributed to increased disease severity in PAD4^f/f^Cre^LysM^ mice. Furthermore, differences in transcriptional profile between PAD4^f/f^Cre^LysM^ and PAD4^−/−^ suggests both intrinsic and extrinsic citrullination can modulate synovial macrophages.

## Materials & Methods

### Mice and models of arthritis

PAD4^−/−^, PAD4^f/f^, and Cre^LysM^ mice were obtained from Jackson Labs. C57Bl/6, PAD4^f/f^Cre^LysM^, and KRNAg^7^ mice were bred in house. STIA was induced at 8–10 weeks using one intravenous injection of 85 mL sera/20 g bodyweight in a final volume of 200mL. STIA was assessed using clinical scoring on a scale of 1–3 per joint, summed over 4 major paw joints. Due to sex bias of arthritis, female mice were used for all studies. Values shown for all groups are mean N=^3^4.

### Administration of PAD inhibitor

BB-Cl-amidine synthesized as described (23) and prepared to 2mg/mL in 25% DMSO in PBS. 100 mL and 2 mL of inhibitor or vehicle only control was administered by intra-peritoneal or intra-articular routes respectively, resulting in a final dose of 0.2 mg I.P or 0.04 mg/joint I.A.

### Flow cytometry

Single-cell suspensions were prepared from blood and synovium as described (24) and stained with Invitrogen eBioscience fixable viability dye and an antibody cocktail to identify myeloid cell populations (CD45^+^, CD11b^+^), neutrophils (Ly6G^+^), monocytes (CD115^+^ or CD64^−^ Ly6c^hi/lo^), macrophage subpopulations (CD64^+^ MHCII^+/−^ CX3CR1^+/−^) whilst excluding lymphocytes CD4^+^, CD8^+^, CD19^+^. Stained cells were fixed with 2% paraformaldehyde. For intracellular staining, cells were permeabilized prior to staining with iNOS and arginase antibodies (both Abcam), as per kit instructions in BD Fixation/Permeabilization solution kit (BD Bioscience). Cells were analyzed using a BD LSRii flow cytometer. Flow cytometry data was analyzed using Flowjo v10. Expression was measured using median fluorescence intensity (MFI). Cell numbers were quantified using 123count eBeads (Thermofisher).

### Serum cytokine analysis

Serum cytokine analysis was carried out using a Luminex200 multiplex, using a Thermofisher Procarta Luminex kit, as per manufacturer instructions.

### Quantification of intracellular citrullination

LC/MS-MS detection of citrullinated peptides was carried out as previously described (25).

### RNA-sequencing

Synovial macrophages were FACSorted into Mac1–4 populations based on expression of CX3CR1 and MHCII using a BD FACSaria. Macrophages were sorted from four 8–10 week old naive PAD4^f/f^LysM-Cre and C57Bl/6 mice, and three PAD4^−/−^ mice. For STIA studies, macrophages were sorted from 4 of each genotype on D7 post-STIA induction, representing peak disease. RNA was extracted using PicoPure RNA Isolation Kits (ThermoFisher). Isolated RNA was sequenced using Clontech SMART-Seq v4 Ultra Low Input RNA Kit for Sequencing (Clontech). Raw sequencing files were demultiplexed using Illumina bcl2fastq and the resulting FASTQ files were trimmed to remove low-quality and short reads (minimum length = 20bp) using trimmomatic [24]. Trimmed reads were then aligned to the mouse reference genome (mm10) using Tophat aligner (26). Resulting BAM alignment files were processed using HTseq (27) to map aligned reads to genes providing raw gene expression counts. Raw gene counts were normalized for depth using Counts Per Million (CPM) normalization and for gene length using Fragments Per Kilobase Million (FPKM) normalization.

Expressed genes were defined by applying a gene expression cutoff on CPM normalized counts as genes with mean expression ^3^ 7 in any subpopulation. FPKM normalized gene expression counts were used to perform dimensionality reduction using Principal Component Analysis (PCA) method. The variance between samples was visualized as a two-dimensional PCA plot using R (version 3.3.1) for all populations. FPKM normalized gene expression counts were used to compute a Pearson’s correlation for all samples which was visualized using heatmaps in R. K-means clustering of C57Bl/6 macrophages was carried out on genes with Log_2_FC>1 across any population using Morpheus. Enriched pathways were generated using Morpheus clustering output compared to total expressed genes in GOrilla (C57Bl6_cluser_pathways sup file).

Differentially expressed genes (DEG) were calculated using DESeq2 in R, and considered significant if Padj was <0.05 (KO_vs_C57Bl6_DESeq sup file, and STIA_KO_Vs_LysMCre_DESeq sup file). For all visualizations and DEG analysis Lyz2 gene was removed to avoid skewing based on Lyz2 expression in Cre^LysM^ mice. From STIA dataset, 1 Mac1 replicate from PAD4^f/f^LysM-Cre and 1 Mac3 replicate from PAD4^−/−^ were removed due to sequencing error. For all other gene figures, figures were plotted using FPKM expression counts in Prism v9. Statistics were calculated using a two-tailed, unpaired T-test. Expressed gene-sets, pathways, and DEGs referenced in this article are listed in the supplementary data tables. Data is available in Gene Expression Omnibus GSE313265.

### Data analysis and statistics

All statistical analysis excluding RNA-seq data was carried out in Prism v9 using two-tailed, unpaired Mann-Whitney or Krukal-Wallis tests (for analysis of more than 2 groups), with Bonferroni correction for multiple comparisons.

## Results

### PAD4 deletion augments synovial macrophage compartment

**Figure 1:**
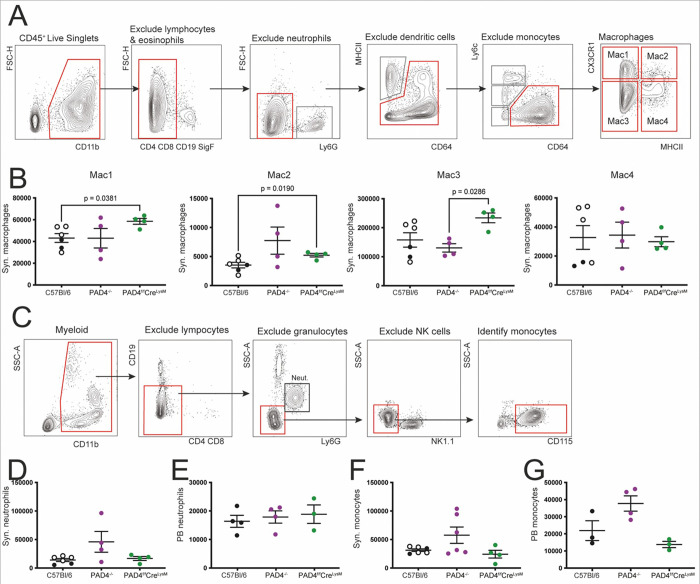
A) Flow cytometry gating strategy that identifies 4 populations of synovial macrophages. B) Numbers of synovial macrophages per joint. Shape of icons indicates different experiment repeats (open vs closed circles) C) gating strategy for peripheral blood myeloid cell populations. D-g) myeloid cell populations in synovium and blood of C57Bl/6, PAD4^−/−^, and PAD4^f/f^ PAD4^f/f^Cre^LysM^ mice in steady state. Figures are mean ± S.E of n^3^4. Significance was calculated using a Mann-Whitney test and considered significant in p<0.05. Significant p values are shown, if not significant values are not shown.

Our prior work has identified four populations of synovial macrophages, with distinct transcriptional profiles (28). Given the divergent roles of these populations in inflammation, we aimed to determine the effect of citrullination by PAD4 on each of these subpopulations. Synovial macrophages were isolated from hind joints of C57Bl/6 mice using flow cytometry staining as CD11b^+^ CD4^−^ CD8^−^ CD19^−^ Ly6G^−^ SSC^lo^ CD64^+^ and subdivided into Mac1: MHCII^−^ CX3CR1^+^, Mac2: MHCII^+^CX3CR1^+^, Mac3: MHCII^−^CX3CR1^−^, and Mac4: MHCII^+^CX3CR1^−^ (Fig. 1a). Macrophage numbers in hind joints of C57Bl/6 mice were compared to mice with global PAD4 deletion (PAD4^−/−^), or LysM specific deletion which targets macrophages, monocytes, and neutrophils (PAD4^f/f^Cre^LysM^) (29) (Fig. 1b). Mac3 and Mac4 were significantly increased in PAD4^f/f^Cre^LysM^ compared to C57Bl/6, whereas numbers from PAD4^−/−^ mice were not significantly affected. No significant changes were observed in Mac1 or Mac2 populations. These data suggest global and cell-type-specific PAD4 deficiency have different effects on synovial macrophages, and that CX3CR1^−^ macrophages are more significantly affected by PAD4. Given both PAD4^−/−^ and PAD4^f/f^Cre^LysM^ would also cause deletion in monocytes and neutrophils, we examined these populations in both synovium and peripheral blood (PB). Surprisingly, numbers of neutrophils or monocytes from PAD4^−/−^ or PAD4^f/f^Cre^LysM^ mice were not significantly altered from WT (Fig. 1c–g).

### PAD4 deletion augments synovial macrophage transcriptional profile

**Figure 2:**
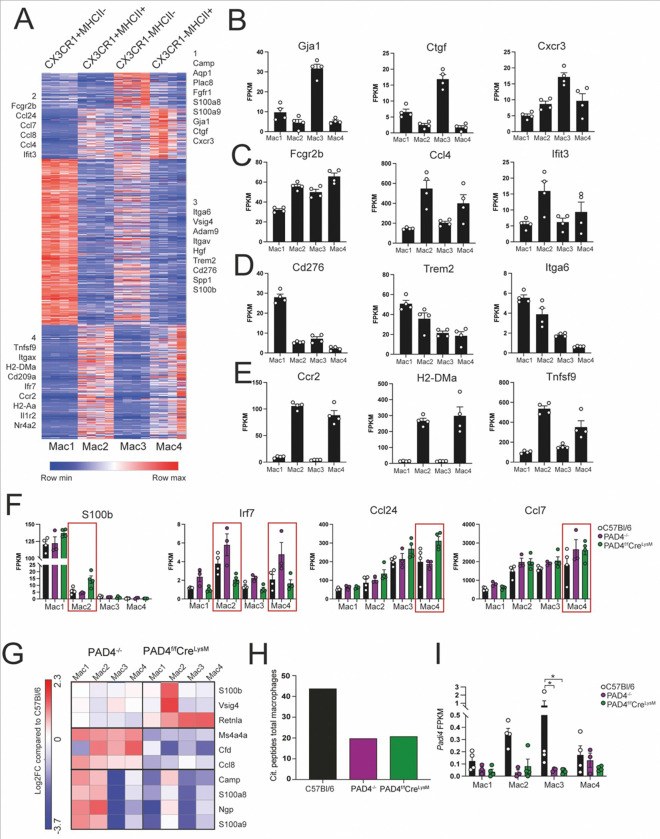
A) Heatmap of K-means clustering of expressed genes from Mac1–4 populations from N=4 C47Bl/6 mice. B-E) Bar charts showing FPKM expression of representative genes from each enriched cluster. F) Expression of representative genes enriched in C57Bl/6 clusters in Mac1–4 from PAD4^−/−^, and PAD4^f/f^Cre^LysM^ mice in steady state. G) Heatmap of significantly different genes expressed in PAD4^−/−^ and PAD4^f/f^Cre^LysM^ compared to C57Bl/6. Heatmap shows mean of N^3^3 replicates, represented as Log2FC compared to C57Bl/6. H) Number of citrullinated peptides detected in total synovial macrophages from C57Bl/6, PAD4^−/−^, and PAD4^f/f^Cre^LysM^ mice. I) Expression of Padi4 gene in Mac1–4 from C57Bl/6, PAD4^−/−^, and PAD4^f/f^Cre^LysM^ mice. Figures are ± S.E. *=p<0.5.

First we characterized macrophage subtypes in WT mice by carrying out k-means clustering on 1131 differentially expressed genes (LogFc > 1), that identified 4 clusters of enriched genes (Fig. 2a). According to our previous work, MHCII^−^ synovial macrophages are embryonic, resident cells in contrast to MHCII^+^ macrophages that are monocyte derived (MDM) (30). In addition, Culemann and colleagues have shown CX3CR1 expression is a marker of resident macrophages in the synovium (31). Therefore, by combining both markers, we aimed to further characterize the heterogeneity in the synovial macrophage compartment. Cluster 1 comprised 96 genes, enriched in Mac3 CX3CR1^−^MHCII^−^ cells. Genes in this cluster included gap junction protein *Gja1*, connective tissue growth factor *Ctgf*, and chemokine *Cxcr3* (Fig. 2b). Within this gene module, enriched pathways included defense response, migration, and proliferation (Fig S1a). Cluster 2 comprise 136 genes, enriched in both MHCII^+^ populations, Mac2 and Mac4 (CX3CR1^+^MHCII^+^, and CX3CR1^−^MHCII^+^) and to a lesser extend Mac3 (CX3CR1^−^MHCII^−^). The wide distribution of expression of these genes indicated shared macrophage functional pathways, which was supported by enrichment of leukocyte chemotaxis, cytokine signaling, and immune response pathways that are characteristic of most macrophages. Enriched genes included Fc receptor *Fcgr2b*, chemokine ligand *Ccl24*, and interferon induced protein *Ifit3* (Fig. 2c). Next, in cluster 3 of 448 genes enriched in Mac1 (CX3CR1^+^MHCII^−^), we observed enrichment in genes associated with resident, homeostatic macrophages. Supported by both our previous findings and others, of MHCII^−^ and CX3CR1^+^ expression denoting embryonic macrophages. Gene enrichment included *Cd276*, an immune checkpoint associated with anti-inflammatory macrophages, *Trem2* which has previously been shown to denote resident synovial macrophages in human RA patients (32), and integrin 6 (*Itga6*) (Fig. 2d). Enriched pathways were also characteristic of resident macrophages, including proliferation, angiogenesis, and cell-matrix adhesion (Fig s1a). Finally, cluster 4 of 451 genes enriched in Mac2 and Mac4, supported the notion that both MHCII^+^ populations were monocyte derived, enriched for monocyte genes such as *Ccr2*, and genes associated with antigen presentation and T-cell activation (*H2-DMa*, *Tnfsf9*), processes typical of pro-inflammatory MDM (Fig. 2e, Fig s1a).

Next, we determined how deletion of *Padi4* influenced these macrophage phenotypes we interrogated transcriptional profiles of each synovial macrophage population from C57Bl/6, alongside PAD4^−/−^, and PAD4^fl^Cre^LysM^. PCA analysis confirmed macrophage population rather than genotype was the main source of variation, with Mac1–4 clustering together with their respective populations (Fig S1b). A heatmap of Pearson’s correlation confirmed these findings, showing a higher level of correlation within macrophage subtype, irrespective of genotype (Fig S1c). These data indicate the loss of *Padi4* expression does not significantly alter macrophage transcriptional profile to the extent phenotype is lost, and instead origin retains influence over profile. However, changes to characteristic genes from each macrophage subtype were observed, including increased expression of *S100b, Irf7, Ccl24, and Ccl7* (Fig. 2f).

We next examined differentially expressed genes in PAD4^−/−^ and PAD4^fl^Cre^LysM^ compared to WT and observed a higher number of genes were significantly altered in expression level (*padj* < 0.05) in macrophages from PAD4^fl^Cre^LysM^ mice compared to WT, than PAD4^−/−^ compared to WT (Fig S1d). In Mac1, 420 genes were significantly changed from PAD4^fl^Cre^LysM^ mice compared to 68 from PAD4^−/−^ mice, in Mac2 this was 144 compared to 14, 56 compared to 29 in Mac3, and 15 compared to 6 in Mac4. These data indicate that in both genotypes putative resident macrophages Mac1 (Cx3CR1^+^ MHCII^−^) were the most significantly impacted by loss of intracellular citrullination. We also identified a cohort of genes enriched in C57Bl/6 WT macrophages (Fig. 2a) that had contrary patterns of expression in PAD4^−/−^ and PAD4^f/f^Cre^LysM^ macrophages (Fig. 2g). In PAD4^f/f^Cre^LysM^ macrophages, inflammatory gene *S100b*, and genes associated with macrophage repair and scavenger function *Vsig4* and *Retnla* were increased in Mac2 compared to WT, whereas these genes were decreased in PAD4^−/−^ Mac2 compared to WT. Conversely, *Ms4a4*, *Cfd*, and Ccl8 were increased in all PAD4^−/−^ macrophages and decreased in all PAD4^f/f^Cre^LysM^ macrophages compared to C57Bl/6 controls. Meanwhile neutrophilic granule protein (*Ngp*), inflammatory s100 protein genes *S100a8* and *S100a9*, and *Camp*, were increased in expression in Mac1 and Mac2 in PAD4^−/−^, and in Mac 2 from PAD4^fl/fl^Cre^LysM^ mice.

To ensure these contrasting results from knock-out models were not down to levels of citrullination, we measured citrullinated peptides by mass spectrometry and observed no differences in levels of citrullination in total synovial macrophages from PAD4^−/−^ compared to PAD4^f/f^Cre^LysM^ mice, however both were reduced compared to C57Bl/6 (Fig. 2h). We also confirmed this was not due to differences in *Padi4* gene expression (Fig. 2i) or a compensatory effect of *Padi2* (Fig S1e). Taken together these data demonstrate the ability for intracellular citrullination by PAD4 to influence key macrophage characteristics, and in addition the divergent outcomes from PAD4 deficiency in global compared to myeloid restricted cells, suggesting both cell intrinsic and extrinsic citrullination can manipulate macrophage function.

### PAD4 deletion in macrophages stimulates immune-metabolic changes

To further investigate the influence of PAD4 on macrophage phenotype, we used immune-metabolic profiling. Macrophages utilize arginine in two distinct pathways, which can profoundly impact the function of the cell. In simplistic terms, metabolism of arginine via iNOS is associated with a classical, pro-inflammatory phenotype, whereas metabolism via arginase is associated with an alternatively activated macrophage phenotype required for wound healing (33). It is possible that intracellular PAD4 activity alter NOS or arginase activity and thus skew the immune-metabolism of the macrophage. To investigate that, we measured intracellular iNOS and arginine as indicators of pro-inflammatory or alternatively activated macrophages states in circulating monocytes and synovial macrophages from C57Bl/6, and in KRNAg^7^mice as a model of systemic, chronic inflammation. We also compared the expression of iNOS and arginase in PAD4^−/−^, and PAD4^f/f^Cre^LysM^ mice.

**Figure 3:**
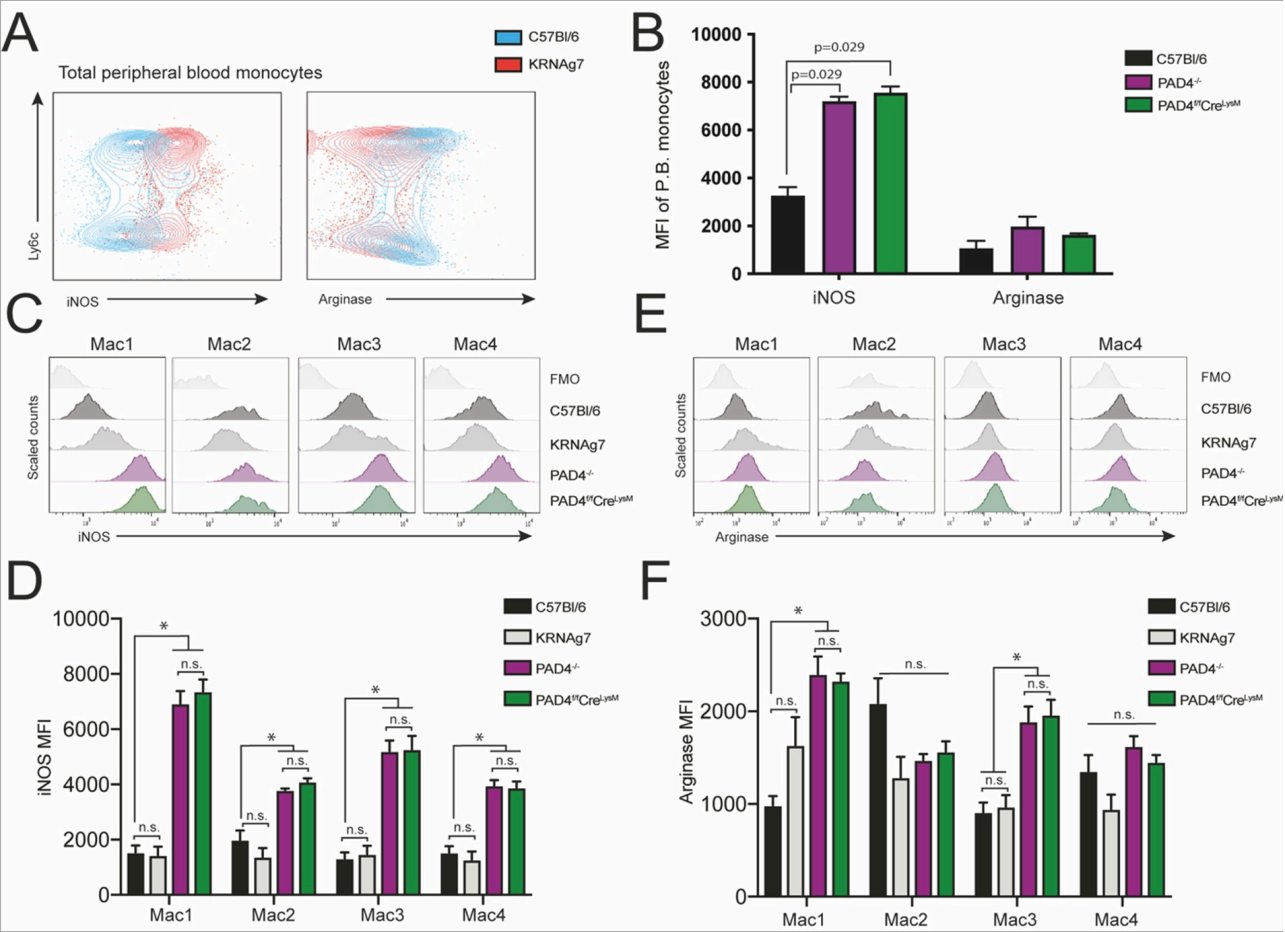
A) Representative flow cytometry image of intracellular staining for iNOS and arginase in peripheral blood monocytes from C57Bl/6 and K/BxN mice. B) Median fluorescent intensity (MFI) of iNOS and arginase staining in peripheral blood monocytes from C57Bl/6, K/BxN, PAD4^−/−^, and PAD4^f/f^Cre^LysM^ mice. C) Representative histograms and D) MFI of iNOS and E-F) arginase staining in synovial macrophages from C57Bl/6, K/BxN, PAD4^−/−^, and PAD4^f/f^Cre^LysM^ mice. All figures are mean ± S.E of n^3^4, statistical significance was calculated using Mann-Whitney testing and considered significant if p<0.05. Statistically significant P values are indicated on figures, or for simplicity asterix are used where *=p<0.5, **=p<0.01, ***=p<0.05.

Representative flow cytometry staining for iNOS and arginase in KRNAg7 mice compared to C57Bl6 confirmed a switch towards increased iNOS and decreased arginase in peripheral blood monocytes (Fig. 3a). Further, PB monocytes from both PAD4^−/−^ and PAD4^f/f^Cre^LysM^ mice had significantly increased staining for iNOS compared to C57Bl/6 controls, however arginase staining was not impacted (Fig. 3b).

In synovial macrophages, iNOS expression was not significantly different between C57Bl/6 and KRNAg^7^ mice or between PAD4^−/−^ and PAD4^fl^Cre^LysM^ in any synovial macrophage population. However, both PAD4-deficient strains had increased iNOS across all macrophages compared to both PAD4-competent phenotypes (*p* < 0.005, *p* < 0.05, *p* < 0.005, *p* < 0.01 for Mac1-Mac4 respectively) (Fig. 3c–d). Arginase expression was more variable across macrophage subpopulations. Expression in both PAD4 knock-out models was comparable in all cells (Fig. 3e–f). In Mac1 and Mac3, PAD4 deletion increased arginase staining significantly compared to C57Bl/6 mice (*p* < 0.01 and *p* < 0.05 respectively), whilst no changes were observed in Mac2 or Mac4. However, no significant differences were observed between synovial macrophages from C57Bl/6 or KRNAg7 mice, suggesting arginase levels are not relevant to synovial macrophages in inflammation. Taken together, these data confirm loss of intracellular citrullination modulates metabolic function of synovial macrophages, and may skew towards a phenotype associated with cells from an inflammatory state, as indicated by increased iNOS expression.

### Cell-type-specific deletion of PAD4 increases severity of inflammatory arthritis

**Figure 4:**
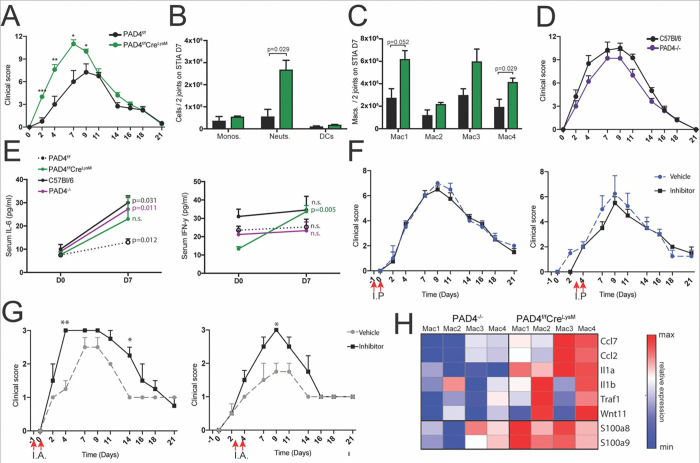
A) Clinical score of STIA, and B) numbers of myeloid cell populations in the synovium of PAD4^f/f^Cre^LysM^ and PAD4^f/f^ mice on day7 of disease. E) Serum cytokine levels in C57Bl/6, PAD4^−/−^, PAD4^f/f^, and PAD4^f/f^Cre^LysM^ in steady state and STIA D7. Significant differences between D0-D7 are indicated. For analysis, each D7 data group was compared to D0 for individual genotypes and cytokines discretely, and therefore no adjustment for multiple comparisons was used. F) Clinical score of STIA in C57Bl/6 mice with/without PAD inhibitor administered prior to or post STIA systemically via IP or G) locally via intra-articular injection. H) A heatmap showing relative expression of genes that were statistically significant (padj <0.05) in at least one group between PAD4^f/f^Cre^LysM^ and PAD4^−/−^ genotypes following DESeq analysis. All figures are mean ± S.E of n^3^4. Significance was calculated using a Mann-Whitney test (for flow cytometry and serum analysis) or unpaired two-tailed t-test for clinical scores. P values were considered significant if p<0.05. Significant p values are shown, or represented by asterix *=p<0.5, **=p<0.01, ***=p<0.05. If not significant values are not shown or are annotated ‘n.s.’.

To determine the effect of PAD4 on the severity of STIA, PAD4^−/−^ and C57Bl/6 controls were administered arthritogenic KBxN serum and clinical disease was assessed for 21 days (Fig. 4a). STIA in PAD4^f/f^Cre^LysM^ mice compared to PAD4^f/f^ controls showed a significant increase in clinical disease score from D2-D9 (Fig. 4a). In the synovial tissue, neutrophils, a known driver of STIA were significantly increased compared to controls (Fig. 4b–c). As observed at steady-state, Mac1 were also significantly increased in PAD4^f/f^Cre^LysM^ mice compared to controls, suggested a pre-disease increase in synovial macrophages could contribute to increased disease severity. Mac4 were not increased in steady-state compared to controls, but also showed a significant increase during STIA. In contrast, STIA severity was not altered in PAD4^−/−^ compared to controls (Fig. 4d). To determine if the increased severity observed for the PAD4^f/f^Cre^LysM^ mice was also evident in serum, we measured serum cytokine levels at D7 of STIA (Fig. 4e). As expected, IL-6 increased in STIA compared to steady-state in all genotypes, although this did not reach significance in the PAD4^f/f^Cre^LysM^ group. However, sera from PAD4^f/f^Cre^LysM^ mice did display a significant increase in IFN-y levels, not observed in any other genotype.

### Local administration of PAD inhibitors increases severity of inflammatory arthritis

In addition to studies using PAD4^−/−^ mice, several experiments have found that the administration of the pan-PAD inhibitors Cl-amidine or BB-Cl-amidine significantly reduces disease severity in inflammatory models including lupus and arthritis. We examined the effect of systemic (intra-peritoneal, I.P.) vs local (intra-articular, I.A.) administration of BB-Cl-amidine, either prior to administration of arthritogenic serum (D-1, D0), or during development of inflammation (D3, D4). Administration of BB-Cl-amidine I.P did not alter severity of STIA either prior to, or during disease, compared to vehicle control (Fig. 4f). Conversely, I.A BB-Cl-amidine increased severity of arthritis administered either prior to or during STIA development (Fig. 4g).

### Synovial macrophages from PAD4^f/f^Cre^LysM^ mice have increased inflammatory genes during STIA

To propose potential mechanisms for the increased severity of STIA in PAD4^f/f^Cre^LysM^ mice not observed in PAD4^−/−^ mice, we carried out bulk RNA-sequencing on Mac1–4 FACSorted from hindjoints of PAD4^f/f^Cre^LysM^ and PAD4^−/−^ mice during peak STIA. DESeq identified significant alterations in gene expression, including enrichment of genes encoding chemoattractants such as *Ccl7*, *Ccl2*, interleukin 1 genes *Il1a/Il1b*, Inflammatory signaling molecules *Traf1*, and *Wnt11*, and inflammatory *S100a8/a9* molecules. These data indicate synovial macrophages from PAD4^f/f^Cre^LysM^ mice exhibit a heightened inflammatory activation state during inflammatory arthritis, compared to PAD4^−/−^ counterparts (Fig. 4h).

## Discussion

In this study, we aimed to determine the effect of citrullination in synovial macrophages on pathology of inflammatory arthritis in mice.

A key finding in these data was the divergence between the effect of global PAD4^−/−^ and cell specific deletion in PAD4^f/f^Cre^LysM^ synovial macrophages. Comparing composition of the synovial macrophage compartment in steady state using subpopulations previously defined, we observed Mac3 and Mac4 in PAD4^f/f^Cre^LysM^ mice were increased compared to PAD4^−/−^ and C57Bl/6 mice, suggesting PAD4 deletion in these cells may have induced proliferation or recruitment of monocyte-derived macrophages from the periphery. Previous studies have shown PAD4 controls transcription factor c-MYC, and deletion of PAD4 in bone marrow progenitors induces proliferation (34). Given c-MYC is also involved in macrophage proliferation (35), expansion of synovial macrophages in PAD4^f/f^Cre^LysM^ mice may use the same mechanism. However, this does not explain differences between numbers of PAD4^f/f^Cre^LysM^ mice and PAD4^−/−^ synovial macrophages, which did not expand.

To confirm these opposing effects were not due to levels of citrullination, we confirmed both PAD4^−/−^ and PAD4^f/f^Cre^LysM^ mice had reduced numbers of intracellular citrullinated peptides, and *Padi4* gene expression compared to C57Bl/6.

Deletion of PAD4 also induced changes in immune-metabolism in synovial macrophages. Metabolism of arginine is mediated by two distinct pathways: in pro-inflammatory macrophages arginine is metabolized by inducible nitric oxide synthase (iNOS) to NO and citrulline, whereas in alternatively activated macrophages it is hydrolyzed by arginase to ornithine and urea. We first confirmed this system in PB monocytes from WT and KRNAg^7^ mice, as a model of systemic inflammation. These PB monocytes had reduced arginase, and increased iNOS compared to controls, as expected. Next, we examined synovial macrophages. Surprisingly, synovial macrophages from KRNAg7 were not significantly different in either iNOS or arginase compared in C57Bl/6 in any subpopulation. This finding contradicts reports that synovial macrophages have increased iNOS in arthritis patients (36). However synovial macrophages from both PAD4^−/−^ and PAD4^f/f^Cre^LysM^ mice had significantly increased iNOS in all subpopulations compared to controls. Arginase was also increased in Mac1 and Mac3 in both genotypes. Under steady state conditions, iNOS is translationally controlled by arginase and so upregulation of both pathways at once is not typical, but has been demonstrated in CD11b+ cells in cryptococcus gattii infection and tumour-associated macrophages in culture (37, 38).

We also examined global transcriptional profiles of PAD4^−/−^ and PAD4^f/f^Cre^LysM^ mice compared to C57Bl/6. PAD4 citrullinates nuclear proteins including histones (1–3) and transcription factors and can therefore influence transcription (7, 8). Interestingly when compared to C57Bl/6, PAD4^f/f^Cre^LysM^ had more total DEG than between PAD4^−/−^ and C57Bl/6, supporting the notion of cell intrinsic and extrinsic mechanisms dependent on citrullination that are capable of modifying gene expression in synovial macrophages. To determine how these transcriptional changes influence inflammatory arthritis, we induced STIA in both models alongside controls. First, we confirmed previous findings that PAD4^−/−^ are equally susceptible to STIA as WT, and no differences in composition of the PB of synovial myeloid compartment were observed. A previous study of STIA in PAD4^−/−^ mice found comparable disease and histological assessment compared to WT, although splenic PAD4 expression may correlate with STIA severity (18, 19). Conversely, PAD4^f/f^Cre^LysM^ mice displayed increased disease severity and immune infiltration compared to PAD4^f/f^ controls, indicating citrullination by PAD4 can also have anti-inflammatory effects. This is supported by the findings of Eghbalzadeh and colleagues, who demonstrated a role for NETs in polarizing a regulatory ‘M2’-like phenotype in macrophages, which was hindered in PAD4^−/−^ mice (39). In addition, transcriptional profiling showed increased expression of chemokines such as *Ccl24*, and pro-inflammatory mediators like *s100b* in macrophages from PAD4^f/f^Cre^LysM^ mice, which could contribute to increased arthritis severity. Only serum from PAD4^f/f^Cre^LysM^ mice displayed a significant increase in IFN-γ on STIA D7 compared to steady-state, in line with our finding that these mice developed more significant disease. Furthermore, interrogation of transcriptional profiles of Mac1–4 from PAD4^f/f^Cre^LysM^ and PAD4^−/−^ mice during STIA also supports the notion that upregulated inflammatory genes in synovial macrophages contributes to heightened arthritis severity. We observed increased expression of chemoattractants *Ccl7* and *Ccl2*, as well as interleukin 1 genes *Il1a/b*, and signaling molecules *Traf1* and *Wnt11* in PAD4^f/f^Cre^LysM^ macrophages during STIA compared to PAD4^−/−^ macrophages.

The findings reported here highlight the importance of elucidating the impact of cell intrinsic versus extrinsic citrullination to explain dichotomy of findings here using and previous studies highlighting an anti-inflammatory effect of global PAD4 inhibition (17, 40–43). Recent understanding of macrophage biology has highlighted the importance of the macrophage niche: neighbouring stromal cells that provide physical and trophic support to macrophages for their survival and proliferation. In the synovium, synovial fibroblasts are likely to act as a supporting niche for synovial macrophages (44) and are susceptible to citrullination induced phenotypic changes (45). Therefore, it is possible that the dichotomy between global and restricted PAD4 deletion may be a result of modulation of the macrophage environment.

## Conclusions

This study demonstrates a previously undocumented anti-inflammatory role for citrullination by PAD4 in synovial macrophages. In addition, the differing effects of global and myeloid specific PAD4 deletion has highlighted the complexity of intracellular citrullination, and the need for greater understanding of citrullination as a mechanism of macrophage modulation.

## Supplementary Material

Supplementary Files

This is a list of supplementary files associated with this preprint. Click to download.

• C57Bl6clusterpathways.xlsx

• C57Bl6expressedgenes.xlsx

• STIAKOVsLysMCreDESeq.xlsx

• KOvsC57Bl6DESeq.xlsx

• SupplementaryFigure1.docx

## Data Availability

The datasets generated during the current study are available in the GEO repository GSE313265 and relevant analysed data is included as supplementary files in this article.

## Abbreviations

PAD4: Peptidylarginine deiminase enzyme 4
STIA: serum transfer induced arthritis
PCA: Principal component analysis
